# Different Levels of Skin Whitening Activity among 3,6-Anhydro-l-galactose, Agarooligosaccharides, and Neoagarooligosaccharides

**DOI:** 10.3390/md15100321

**Published:** 2017-10-20

**Authors:** Ji Hye Kim, Eun Ju Yun, Sora Yu, Kyoung Heon Kim, Nam Joo Kang

**Affiliations:** 1 School of Food Science and Biotechnology, Kyungpook National University, Daegu 41566, Korea; friend8201@naver.com; 2 Korean Medicine Application Center, Korea Institute of Oriental Medicine, Daegu 41062, Korea; 3 Department of Biotechnology, Graduate School, Korea University, Seoul 02841, Korea; ejyun0824@gmail.com (E.J.Y.); sora90715@korea.ac.kr (S.Y.)

**Keywords:** 3,6-anhydro-l-galactose, agarooligosaccharides, neoagarooligosaccharides, skin whitening, α-melanocyte-stimulating hormone, melanogenesis

## Abstract

3,6-Anhydro-l-galactose (AHG), a major monomeric constituent of red macroalgae (*Rhodophyta*), was recently reported to possess skin whitening activity. Moreover, AHG-containing oligosaccharides, such as agarooligosaccharides (AOSs) and neoagarooligosaccharides (NAOSs), have various physiological activities, including anti-inflammatory, antioxidant, and skin moisturizing effects. In this study, AHG and NAOSs were produced from agarose by enzymatic reactions catalyzed by an endo-type β-agarase, an exo-type β-agarase, and a neoagarobiose hydrolase. In a cell proliferation assay, AHG, AOSs, and NAOSs at 12.5, 25, and 50 μg/mL concentrations did not exhibit cytotoxicity toward murine B16 melanoma cells or human epidermal melanocytes. In an in vitro skin whitening activity assay of AHG, AOSs, and NAOSs at 50 μg/mL, AHG showed the highest skin whitening activity in both murine B16 melanoma cells and human epidermal melanocytes; this activity was mediated by the inhibition of melanogenesis. Neoagarotetraose and neoagarohexaose also exhibited in vitro skin whitening activity, whereas neoagarobiose and AOSs with degrees of polymerization of 3 (agarotriose), 5 (agaropentaose), and 7 (agaroheptaose) did not. Therefore, AHG is responsible for the skin whitening activity of agar-derived sugars, and the structural differences among the AHG-containing oligosaccharides may be responsible for their different skin whitening activities.

## 1. Introduction

**Figure 1:**
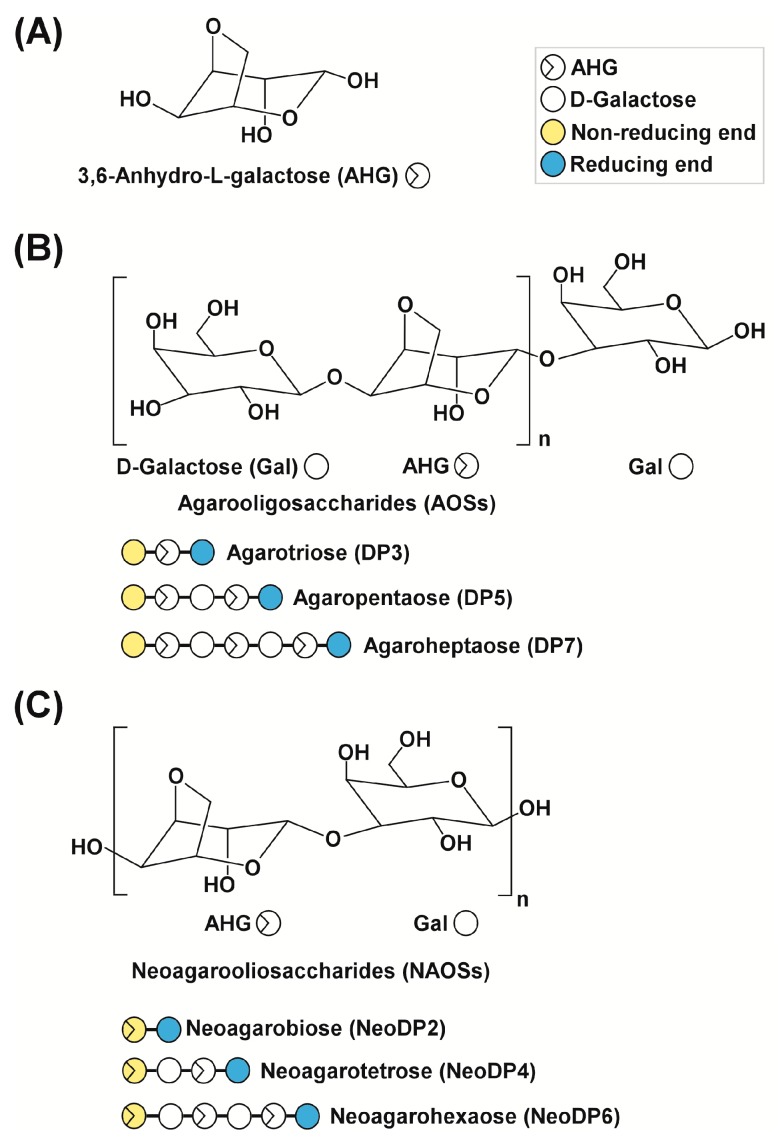
Chemical structures of agarose-derived oligosaccharides. (**A**) 3,6-Anhydro-l-galactose (AHG); (**B**) agarooligosaccharides (AOSs), including agarotriose (DP3), agaropentaose (DP5), and agaroheptaose (DP7); and (**C**) neoagarooligosaccharides (NAOSs), including neoagarobiose (NeoDP2), neoagarotetraose (NeoDP4), and neoagarohexaose (NeoDP6). The non-reducing end of AOSs is a d-galactose (Gal) unit, whereas that of NAOSs is an AHG unit. DP3 is the smallest odd-numbered AOS, and NeoDP2 is the smallest even-numbered NAOS.

Melanogenesis, the process by which melanin is produced, is primarily regulated by ultraviolet (UV) light [1]. In response to UV light, various stimulators, such as α-melanocyte-stimulating hormone (α-MSH), are produced locally in the skin [2,3]. α-MSH stimulates the activity or expression of tyrosinase (EC 1.14.18.1), which catalyzes the rate-limiting step in melanogenesis [4,5]. The upregulation of tyrosinase enhances the conversion of l-tyrosine to l-3,4-dihydroxyphenylalanine (l-DOPA) by hydroxylation, resulting in a rapid increase in melanin production [6]. In addition, two key substrates, l-tyrosine and l-DOPA, reportedly modulate melanin synthesis as hormone-like regulators [7], which is accompanied by hyperpigmentation, in cultured melanin-producing cells such as a murine melanoma cell line, B16F10 and human epidermal melanocytes, HEMs [6,7,8]. Therefore, melanogenesis can be modulated to prevent skin pigmentation, including freckles, melasma, and age spots [1]. According to previous studies, seaweeds (marine macroalgae) are promising sources of novel melanogenesis inhibitors because of their skin whitening effects [9,10,11]. This biological effect of marine macroalgae is likely the result of macroalgae-derived sugars [9,10,11]. Previously, we reported for the first time that 3,6-anhydro-l-galactose (AHG) (Figure 1A), the key monomeric sugar of red macroalgae, exerts a marked anti-melanogenic effect [12].

To produce AHG, the liquefaction of agarose, which is the main carbohydrate of red macroalgae, using an acid or endo-type β-agarase and enzymatic saccharification is necessary [12]. First, agarose is depolymerized into agarooligosaccharides (AOSs), which have d-galactose (Gal) at the non-reducing end, or neoagarooligosaccharides (NAOSs), which have AHG at the non-reducing end, by liquefaction via an acid or endo-type β-agarase, respectively (Figure 1B,C) [12,13]. The AOSs or NAOSs are then enzymatically hydrolyzed into neoagarobiose (NeoDP2) by an exo-type β-agarase. Finally, NeoDP2 is further hydrolyzed into AHG and Gal by α-neoagarobiose hydrolase (NABH) [14].

The anti-melanogenic effect of AHG and NAOSs has been reported by others [14,15,16]. NeoDP2 exerts an anti-melanogenic effect and has low cytotoxicity in B16 murine melanoma cells [16]. NAOSs, including neoagarotetraose (NeoDP4) and neoagarohexaose (NeoDP6), also inhibit melanin synthesis and cellular tyrosinase activity in B16F10 cells [15]. These bioactivities of NAOSs are likely due primarily to the presence of an AHG unit at the non-reducing end, as reported previously by our group [14]. However, the anti-melanogenic activity of AHG and NAOSs has not been performed individually and were not compared at the same time. In addition, although AOSs also contain AHG, together with Gal, as their major sugar units, the anti-melanogenic activity of AOSs has not been reported to date. Furthermore, the biological effects of AHG, AOSs, and NAOSs have not been examined in human cells, such as HEMs, indicating that their skin whitening activity still needs to be determined.

In this study, we investigated whether AHG and AHG-containing AOSs and NAOSs exert anti-melanogenic activity in murine B16F10 melanoma cells and HEMs. To compare the anti-melanogenic activity of AHG, AOSs, and NAOSs, their effects on α-MSH-induced melanin production were investigated in B16F10 cells and HEMs.

## 2. Results and Discussion

### 2.1. Identification of AHG, NAOSs, and AOSs by Liquid Chromatography Hybrid Ion Trap Time-of-Flight Mass Spectrometry (LC/MS-IT-TOF) Analysis

**Figure 2:**
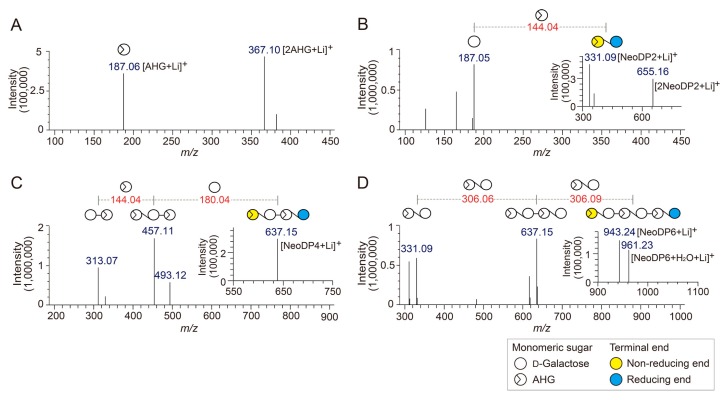
Identification of 3,6-anhydro-l-galactose (AHG), neoagarobiose (NeoDP2), neoagarotetraose (NeoDP4), and neoagarohexaose (NeoDP6) by LC/MS-IT-TOF analysis. Mass spectrum of (**A**) AHG and tandem mass spectra of (**B**) NeoDP2, (**C**) NeoDP4, and (**D**) NeoDP6. The insets in **B**, **C**, and **D** show the mass spectra of the respective neoagarooligosaccharides.

To determine the exact masses of AHG, NeoDP2, NeoDP4, and NeoDP6 generated from reactions catalyzed by Aga16B, Aga50D, and *Sd*NABH, LC/MS-IT-TOF analysis was performed. AHG showed major peaks at *m*/*z* 187.06 and 367, which correspond to the exact masses of the lithium adduct ions of hydrated AHG and hydrated AHG dimer, respectively (Figure 2A). These results suggested that AHG exists mainly in its hydrated form under aqueous condition, as the exact mass of AHG in water is 180, not 162 Da (Figure 2A) [17].

**Figure 3:**
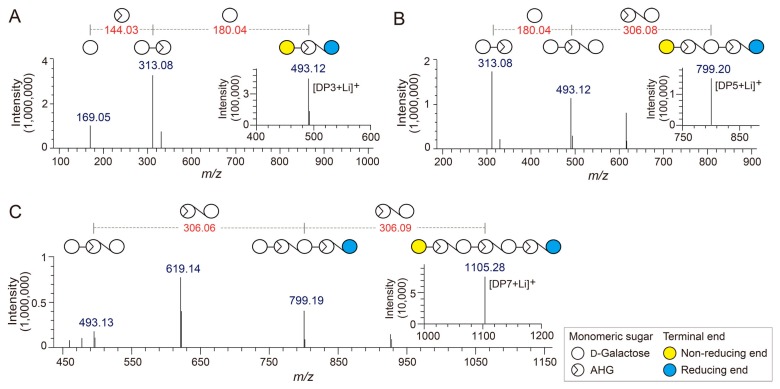
Identification of agarotriose (DP3), agaropentaose (DP5), and agaroheptaose (DP7) by LC/MS-IT-TOF analysis. Tandem mass spectra of (**A**) DP3, (**B**) DP5, and (**C**) DP7. The insets in A, B, and C show the mass spectra of the respective agarooligosaccharides.

NeoDP2, NeoDP4, and NeoDP6 showed major peaks at *m*/*z* 331.09, 637.15, and 943.24, respectively, which correspond to the exact masses of the lithium adduct ions of these NAOSs (Figure 3B–D). The chemical structures of NeoDP2, NeoDP4, and NeoDP6 were verified by tandem mass spectrometry (MS/MS) analysis. Specifically, the fragment ions produced from the precursor ions of NeoDP2, NeoDP4, and NeoDP6 demonstrated that these NAOSs consist of AHG and Gal units (Figure 3B–D).

**Figure 4:**
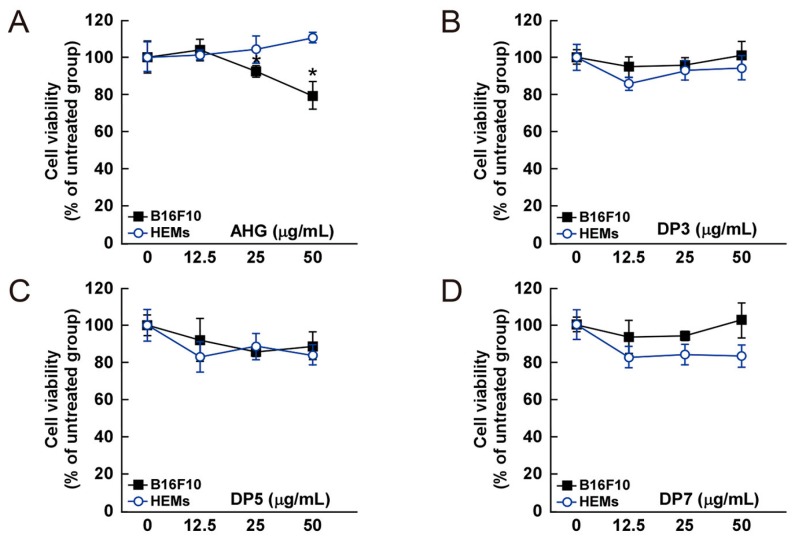

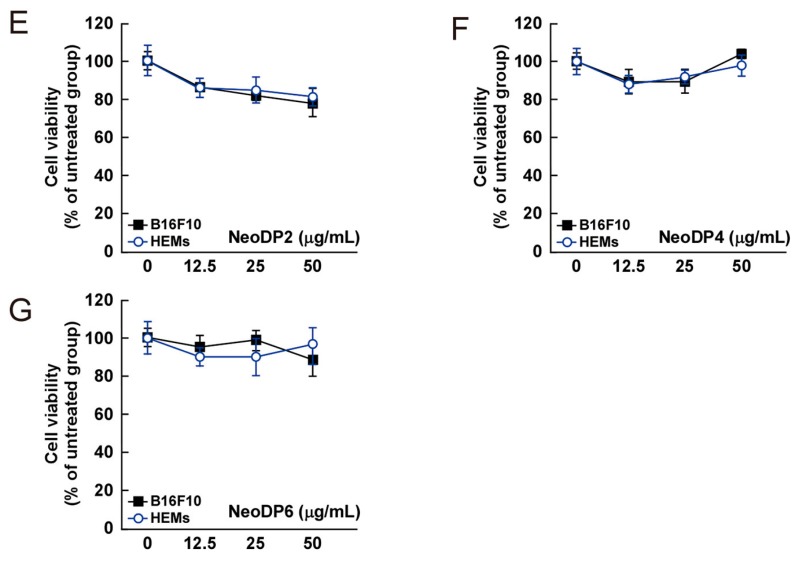
Effect of 3,6-anhydro-l-galactose (AHG) and AHG-containing agarooligosaccharides and neoagarooligosaccharides on the proliferation of B16F10 cells and human epidermal melanocytes (HEMs). (**A**) AHG, (**B**) agarotriose (DP3), (**C**) agaropentaose (DP5), (**D**) agaroheptaose (DP7), (**E**) neoagarobiose (NeoDP2), (**F**) neoagarotetraose (NeoDP4), and (**G**) neoagarohexaose (NeoDP6) at concentrations up to 50 μg/mL exhibited no cytotoxic effects on day 3. The cytotoxicity of each sugar sample was evaluated by MTT assay, as described in the Experimental Section. Data are presented as the means ± SD of three independent experiments. Asterisks indicate a significant difference (* *p* < 0.05) compared with the untreated group.

The chemical structures of the odd-numbered AOSs (DP3, DP5, and DP7) were also determined by LC/MS-IT-TOF analysis (Figure 3). DP3, DP5, and DP7 showed major peaks at *m*/*z* 493.12, 799.20, and 1105.28, respectively, which correspond to the exact masses of the lithium adduct ions of these AOSs (Figure 4A–C). The fragment ions generated from the precursor ions of DP3, DP5, and DP7 demonstrated that these AOSs also consist of AHG and Gal units, similar to the NAOSs (Figure 3B–D).

### 2.2. Cytotoxicity of AHG, NAOSs, and AOSs

The cytotoxic effect of AHG, DP3, DP5, DP7, NeoDP2, NeoDP4, and NeoDP6 was evaluated by MTT assay in melanin-producing B16F10 cells and HEMs. At 25–50 μg/mL, none of the samples exerted a cytotoxic effect on either cell line (Figure 4).

### 2.3. Effects of AHG, NAOSs, and AOSs on Melanogenesis in Murine B16 Melanoma Cells and Human Epidermal Melanocytes

**Figure 5:**
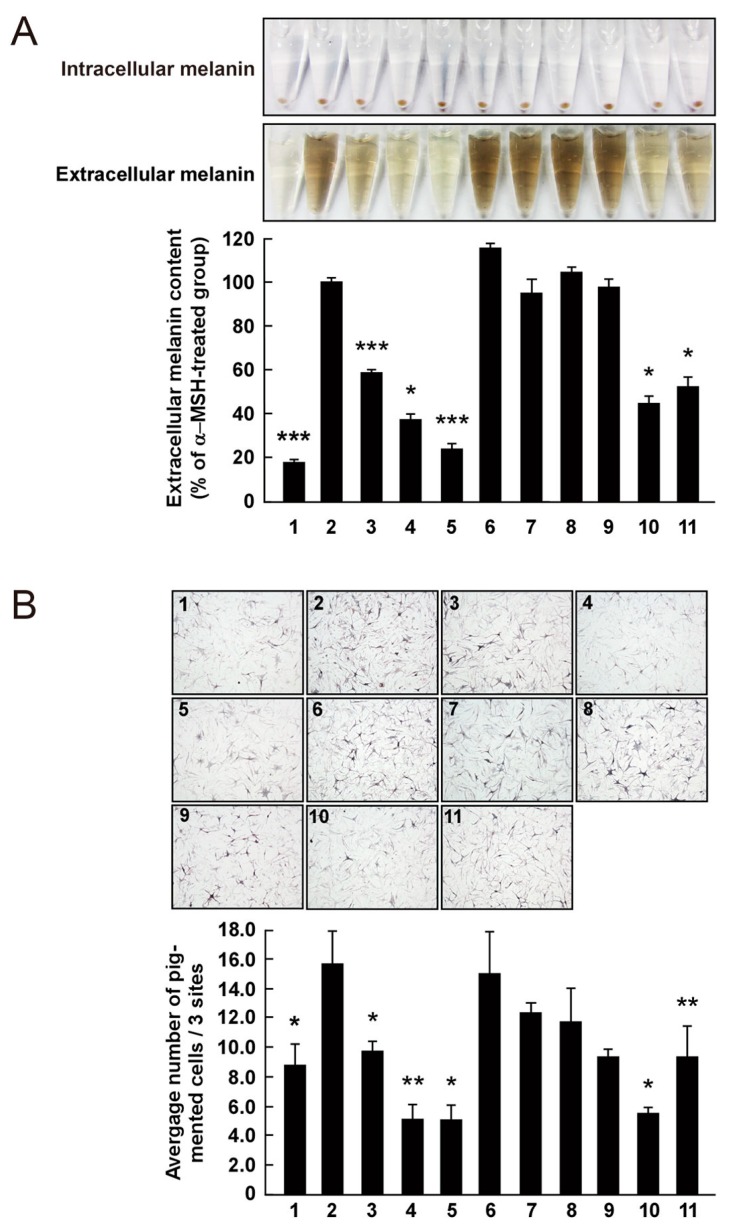
Effect of 3,6-anhydro-l-galactose (AHG) and AHG-containing agarooligosaccharides (AOSs) and neoagarooligosaccharides (NAOSs) on melanogenesis in (**A**) murine B16 melanoma (B16F10) cells and (**B**) human epidermal melanocytes (HEMs). (**A**) AHG and NAOSs inhibited α-melanocyte-stimulating hormone (α-MSH)-induced melanin production in B16F10 cells, but AOSs did not. B16F10 cells were pretreated with each sugar sample for 1 h before exposing to α-MSH (100 nM). Three days later, a melanin content was assayed, as described in the Experimental Section. 1, untreated control cells; 2, α-MSH; 3, α-MSH and 25 μg/mL arbutin; 4, α-MSH and 50 μg/mL arbutin; 5, α-MSH and 50 μg/mL AHG; 6, α-MSH and 50 μg/mL agarotriose (DP3); 7, α-MSH and 50 μg/mL agaropentaose (DP5); 8, α-MSH and 50 μg/mL agaroheptaose (DP7); 9, α-MSH and 50 μg/mL neoagarobiose (NeoDP2); 10, α-MSH and 50 μg/mL neoagarotetraose (NeoDP4); and 11, α-MSH and 50 μg/mL neoagarohexaose (NeoDP6). All photographs were taken at the same magnification using a digital camera. The melanin level in the culture medium was analyzed by measuring the absorbance at 495 nm. Data are presented as the means ± SD of three independent determinations. Asterisks indicate a significant difference (*, *p* < 0.05; ***, *p* < 0.001) compared with the α-MSH-treated group. (**B**) AHG and NAOSs inhibited α-MSH-induced melanin production in HEMs, but AOSs did not. HEMs were pretreated with each sugar sample for 1 h before being exposed to α-MSH (100 nM). Three days later, Fontana-Masson staining was performed to visualize pigmented melanocytes, as described in the Experimental Section. 1, Untreated control cells; 2, α-MSH; 3, α-MSH and 25 μg/mL arbutin; 4, α-MSH and 50 μg/mL arbutin; 5, α-MSH and 50 μg/mL AHG; 6, α-MSH and 50 μg/mL DP3; 7, α-MSH and 50 μg/mL DP5; 8, α-MSH and 50 μg/mL DP7; 9, α-MSH and 50 μg/mL NeoDP2; 10, α-MSH and 50 μg/mL NeoDP4; and 11, α-MSH and 50 μg/mL NeoDP6. All photographs were taken at the same magnification. Pictures are representative of three independent experiments that gave similar results. Pigmented melanocytes were enumerated at three randomly selected sites under a microscope at 400× magnification and are expressed relative to the untreated control. Data are presented as the means ± SD of three independent experiments. Asterisks indicate a significant difference (*, *p* < 0.05; **, *p* < 0.01) compared with the α-MSH-treated group.

The effect on melanogenesis of AHG, NAOSs, and AOSs at non-cytotoxic concentrations was determined by measuring α-MSH-induced intracellular and extracellular melanin levels in B16F10 cells. AHG, NeoDP4, and NeoDP6 markedly reduced melanin secretion (Figure 5A). Their effect was similar to that of arbutin, an established whitening agent. NeoDP4 is reportedly a stronger whitening agent than NeoDP2 and NeoDP6 [15]. Indeed, in this study, NeoDP4 at 50 μg/mL was the most effective whitening agent, followed by NeoDP6 and NeoDP2. In contrast, the odd-numbered AOSs (DP3, DP5, and DP7) at 50 μg/mL did not affect α-MSH-induced melanin production in B16F10 cells.

To the best of our knowledge, there are no reports on the anti-melanogenic activity of AHG and AHG-containing AOSs and NAOSs in HEMs. Therefore, HEMs were treated with each sugar (i.e., AHG, DP3, DP5, DP7, NeoDP2, NeoDP4, or NeoDP6), and intracellular melanin accumulation was visualized by Fontana-Masson staining. As shown in Figure 5B, AHG and arbutin significantly reduced the intracellular melanin content in HEMs. All of the even-numbered NAOSs (NeoDP2, NeoDP4, and NeoDP6) showed a moderate anti-melanogenic effect on intracellular melanin levels in HEMs (Figure 5B). However, none of the odd-numbered AOSs (DP3, DP5, and DP7) reduced melanin production (Figure 5B). These findings suggest that AHG exerts the strongest inhibitory effect against melanin synthesis stimulated by α-MSH in both murine melanoma cells and HEMs. Interestingly, regardless of the presence of AHG as a component, the AOSs and NAOSs exhibited different anti-melanogenic effects. The main difference between these two types of oligosaccharides is that the non-reducing end of NAOSs contains AHG, whereas that of AOSs contains Gal [12]. Moreover, AOSs and NAOSs have different biosynthetic pathways: AOSs are produced mainly by the acid hydrolysis of agarose, whereas NAOSs are produced by the enzymatic hydrolysis of agarose or AOSs by β-agarases. On this basis, we suggest that these structural differences may be responsible for the different anti-melanogenic activity of AOSs and NAOSs in murine melanoma cells and HEMs.

## 3. Experimental Section

### 3.1. Enzymatic Production of AHG, NAOSs, and AOSs

**Figure 6:**
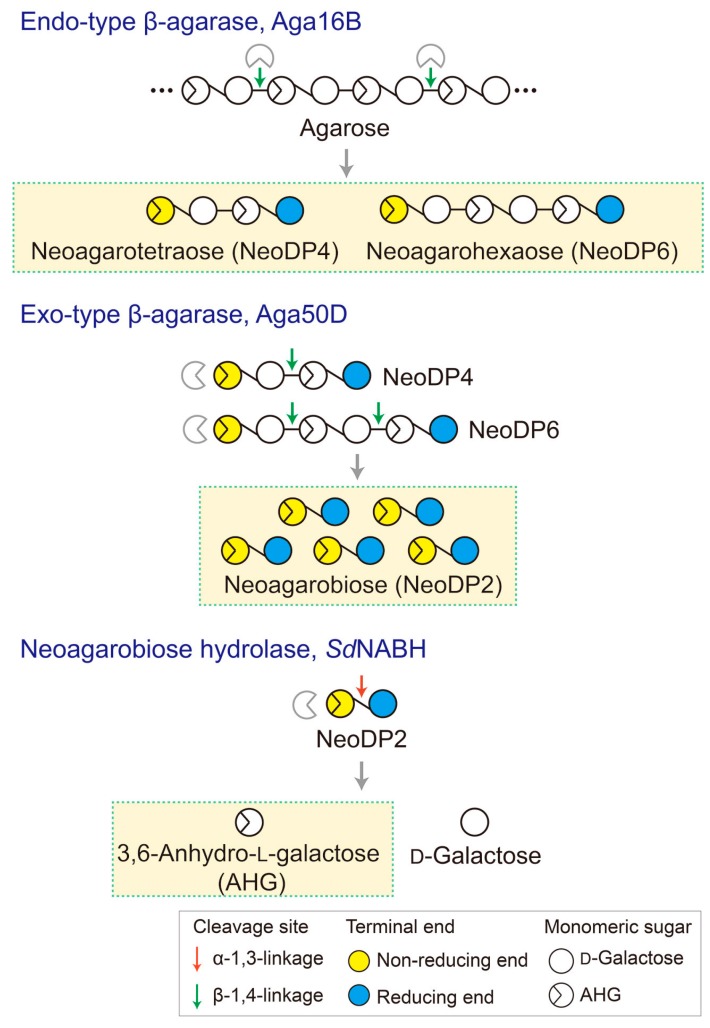
Schematic diagram of the production of 3,6-anhydro-l-galactose (AHG), neoagarobiose (NeoDP2), neoagarotetraose (NeoDP4), and neoagarohexaose (NeoDP6) by enzymatic reactions catalyzed by Aga16B, Aga50D, and *Sd*NABH. First, Aga16B produces NeoDP4 and NeoDP6 from agarose by endolytic cleavage of agarose. Aga50D produces NeoDP2 from the reaction products derived from Aga16B (mainly NeoDP4 and NeoDP6). Finally, *Sd*NABH produces AHG and d-galactose from the reaction products derived from Aga16B and Aga50D (mainly NeoDP2).

To generate AHG, NeoDP2, NeoDP4, and NeoDP6 from agarose, three enzymes involved in the β-agarase system of *Saccharophagus degradans* 2-40^T^, Aga16B (an endo-type β-agarase), Aga50D (an exo-type β-agarase), and *Sd*NABH (an NABH), were used [18,19,20]. First, NeoDP4 and NeoDP6 were produced by the enzymatic reaction of Aga16B with agarose. Aga16B was incubated in 20 mM Tris-HCl buffer (pH 7.0) containing 5% (*w*/*v*) agarose with shaking at 200 rpm for 12 h at 55 °C (Figure 6). Aga50D was then added to the reaction products derived from Aga16B (mainly NeoDP4 and NeoDP6), and the mixture was incubated with shaking at 200 rpm for 12 h at 25 °C to produce NeoDP2 (Figure 6). Finally, *Sd*NABH was added to the reaction products derived from Aga16B and Aga50D (mainly NeoDP2), and the mixture was incubated with shaking at 200 rpm for 12 h at 30 °C to produce AHG and Gal (Figure 6).

Next, AHG, NeoDP2, NeoDP4, and NeoDP6 were purified by affinity chromatography using a silica gel column (internal diameter (I.D.) 4 × 100 cm, 70–230 mesh ASTM; Merck, Darmstadt, Germany) with dichloromethane:methanol:water (78:20:2, *v*/*v*/*v*) as the mobile phase [14], or by size-exclusion chromatography using a Bio-Gel P-2 column (I.D. 2.1 × 35.8 cm; Amersham Biosciences, Piscataway, NJ, USA) with water as the mobile phase [14]. The odd-numbered AOSs, agarotriose (DP3), agaropentaose (DP5), and agaroheptaose (DP7), were purchased from Carbosynth (Compton, UK).

### 3.2. Analysis of AHG, NAOSs, and AOSs by LC/MS-IT-TOF

To analyze AHG, NAOSs, and AOSs, LC/MS-IT-TOF (Shimadzu, Kyoto, Japan) using a Hypercarb Porous Graphitic Carbon LC column (100 × 2.1 mm, packed with 3 μm particles; Thermo Fisher Scientific, Waltham, MA, USA) was performed. One microliter of each sugar sample (~1 mg/mL) was injected onto the column. During the analysis, the temperatures of the autosampler and LC column were maintained at 10 °C and 70 °C, respectively. The mobile phase comprised 25 μM lithium chloride and acetonitrile, and 0–80% gradient elution was performed at a flow rate of 0.2 mL/min for 41 min. Electrospray ionization was operated in the positive-ion mode. Source-dependent parameters were set as follows: nebulizing gas flow, 1.5 L/min; interface voltage, 4.5 kV; detector voltage, 1.65 kV; curved desolvation line temperature, 200 °C; and heat-block temperature, 200 °C. The mass scan range was *m*/*z* 100–1200. To obtain MS/MS data from the precursor ions, the collision-induced dissociation (CID) parameters, CID energy, collision gas parameter, and frequency were set at 50%, 50%, and 45 kHz, respectively. Raw LC/MS-IT-TOF data were processed using LabSolutions LCMS software (version 3.8; Shimadzu, Kyoto, Japan).

### 3.3. Materials for Measuring Skin Whitening Activity

Dulbecco’s modified Eagle’s medium (DMEM), penicillin-streptomycin, and 0.5% trypsin-ethylenediaminetetraacetic acid were obtained from Gibco (Auckland, New Zealand). Medium 254 and human melanocyte growth supplement (HMGS) were purchased from Cascade Biologics (Portland, OR, USA). Fetal bovine serum (FBS), α-MSH, arbutin, 3-(4,5-dimethylthiazol-2-yl)-2,5-diphenyltetrazolium bromide (MTT), dimethyl sulfoxide, and gelatin were purchased from Sigma-Aldrich (St. Louis, MO, USA). Skimmed milk was purchased from MB Cell (Los Angeles, CA, USA).

### 3.4. Cell Culture

The murine melanoma cell line B16F10 was obtained from the Korean Cell Line Bank (Seoul, Korea) and cultured in DMEM supplemented with 10% FBS and 1% penicillin-streptomycin at 37 °C in a humidified atmosphere containing 5% CO_2_. HEMs, derived from moderately pigmented neonatal foreskin, were purchased from Cascade Biologics. They were cultured in Medium 254 supplemented with HMGS at 37 °C in a humidified atmosphere containing 5% CO_2_.

### 3.5. Cell Proliferation Assay

To estimate the possible cytotoxicity of each sugar sample tested in this study, cell proliferation was determined by MTT assay. B16F10 cells (1 × 10^4^/well) or HEMs (7 × 10^4^/well) were seeded in 96-well plates with culture media at 37 °C in a 5% CO_2_ incubator. After culturing for 72 h, 20 μL of MTT solution were added to each well. The cells were then incubated for 2 h at 37 °C in a CO_2_ incubator, and the absorbance at 570 nm of the cell culture was measured.

### 3.6. Measurement of Extracellular Melanin Levels

Melanin levels were measured using a method described previously, with slight modifications [21]. In brief, a confluent monolayer of B16F10 melanoma cells was trypsinized, and 1 × 10^4^ viable cells suspended in 1 mL of 10% FBS-containing DMEM were added to each well of a 12-well plate. The plates were incubated for 24 h at 37 °C in a humidified atmosphere containing 5% CO_2_. Subsequently, the cells were pretreated with each sugar sample for 1 h at 37 °C before being exposed to 100 nM α-MSH. The cells were harvested three days later. After treatment, the media were collected and the levels of melanin were determined by measuring the absorbance at 495 nm using an ELISA reader (Tecan Sunrise-Basic; Tecan, Gröding, Austria).

### 3.7. Fontana-Masson Staining

Intracellular melanin accumulation was visualized by a slightly modified version of previously described Fontana-Masson staining procedures [22]. HEMs were seeded onto gelatin-coated glass coverslips in each well of a 48-well plate and incubated for 24 h. Then, they were pretreated with each sugar sample for 1 h at 37 °C before being exposed to 100 nM α-MSH for three days. After incubation, the cells were fixed in 100% ethanol for 30 min at room temperature and stained for melanin using a Fontana-Masson staining kit from American MasterTech Scientific (Lodi, CA, USA), in accordance with the manufacturer’s instructions. In brief, cells were stained with ammoniacal silver for 60 min at 60 °C, and incubated in 0.1% gold chloride for 15 min at room temperature followed by 5% sodium thiosulfate for 5 min at room temperature. The coverslips were rinsed in phosphate-buffered saline and the cells were observed and photographed using a microscope (Eclipse 80i, Nikon Instruments, Tokyo, Japan). The images were analyzed using NIS-Elements 3.0 software (Nikon Instruments, Tokyo, Japan).

### 3.8. Statistical Analyses

All experiments were conducted in triplicate. The data are expressed as mean ± standard deviation (SD), and were analyzed using Student’s *t*-test. In all analyses, probability values of *p* < 0.05, *p* < 0.01, and *p* < 0.001 were used as the criteria for statistical significance.

## 4. Conclusions

The present comparative study of AHG and AHG-containing AOSs and NAOSs was performed to investigate their anti-melanogenic effect. AHG exhibited the strongest anti-melanogenic activity, and the even-numbered NAOSs exerted moderate effect on both B16F10 cells and HEMs. However, although AOSs also contain AHG, only AHG itself or NOASs inhibited α-MSH-induced melanin production in murine B16F10 cells and HEMs. Taken together, these results suggest that the structural differences in the compounds evaluated in this study are responsible for their different anti-melanogenic activity. In addition, AHG, which is a key component of agarose-derived oligosaccharides, has potential as a novel skin whitening agent.
